# Monitoring the prevalence of viable and dead cariogenic bacteria in oral specimens and *in vitro* biofilms by qPCR combined with propidium monoazide

**DOI:** 10.1186/1471-2180-13-157

**Published:** 2013-07-13

**Authors:** Ai Yasunaga, Akihiro Yoshida, Kazumasa Morikawa, Kenshi Maki, Suguru Nakamura, Inho Soh, Shuji Awano, Toshihiro Ansai

**Affiliations:** 1 Division of Community Oral Health Development, Kyushu Dental University, 2-6-1 Manazuru, Kokurakita-ku Kitakyushu 803-8580, Japan; 2 Division of Developmental Stomatognatic Function Science, Kyushu Dental University, Kitakyushu 803-8580, Japan

**Keywords:** Dental caries, Dental plaque, Biofilm, Propidium monoazide (PMA), Quantification, qPCR (Real-time PCR), *Streptococcus mutans*, *Streptococcus sobrinus*, Viability

## Abstract

**Background:**

*Streptococcus mutans* and *Streptococcus sobrinus* are associated with the development of dental caries in humans. However, previous diagnostic systems are unsuitable for monitoring viable cell numbers in oral specimens. Assessing the relationship between the numbers of viable and dead bacterial cells and oral status is important for understanding oral infectious diseases. Propidium monoazide (PMA) has been reported to penetrate dead cells following membrane damage and to cross-link DNA, thereby inhibiting DNA amplification. In the present study, we established an assay for selective analysis of two viable human cariogenic pathogens, *S. mutans* and *S. sobrinus*, using PMA combined with real-time PCR (PMA-qPCR).

**Results:**

We designed species-specific primer sets for *S. mutans* and *S. sobrinus*, generated standard curves for measuring cell numbers, and evaluated the dynamic range of the assay. To determine the effectiveness of the assay, PMA was added to viable and autoclave-killed cell mixtures. PMA treatment effectively prevented DNA amplification from dead cells. No amplification of DNA from dead cells was observed in these organisms. In addition, we applied this assay to analyze viable cell numbers in oral specimens. A significant correlation was found between the number of viable *S. mutans* cells in saliva and that in plaque among caries-free patients, whereas no correlation was observed between saliva and carious dentin. The total and viable cell numbers in caries-positive saliva were significantly higher than those in caries-free saliva. Finally, we analyzed the usefulness of this assay for *in vitro* oral biofilm analysis. We applied PMA-qPCR for monitoring viable *S. mutans* cell numbers *in vitro* in planktonic cells and oral biofilm treated with hydrogen peroxide (H_2_O_2_). In planktonic cells, the number of viable cells decreased significantly with increasing H_2_O_2_ concentration, whereas only a small decrease was observed in biofilm cell numbers.

**Conclusions:**

PMA-qPCR is potentially useful for quantifying viable cariogenic pathogens in oral specimens and is applicable to oral biofilm experiments. This assay will help to elucidate the relationship between the number of viable cells in oral specimens and the oral status.

## Background

Dental caries represents one of the most common infectious diseases afflicting humans [1]. Of the mutans group of streptococci, *Streptococcus mutans* (serotype *c*, *e*, *f*, and *k* mutans streptococci) and *Streptococcus sobrinus* (serotype *d* and *g* mutans streptococci), which are Gram-positive oral commensal species, are strongly implicated as etiological agents associated with human dental caries. Previous investigations have reported that *S. sobrinus* has a higher acidogenic capacity compared with *S. mutans*, and the prevalence of *S. sobrinus* is more closely associated with high caries activity than is that of *S. mutans*[2,3]. These studies suggest the importance of the diagnoses of infection by these organisms. Previously, several studies have reported methods for diagnosis of these organisms [4,5]. However, DNA-based detection and quantification of specific bacteria cannot distinguish between live and dead bacteria. Bacterial DNA is degraded after the loss of cell viability; thus, the remaining DNA of already dead bacteria can still act as a template DNA for PCR. Consequently, DNA-based detection systems overestimate the cell population. However, we have not differentiated live and dead bacteria within the context of diagnosis of oral infectious diseases, including dental caries. In the present study, we successfully developed and evaluated a discriminative method between live and dead bacteria for the human cariogenic pathogens *S. mutans* and *S. sobrinus* using propidium monoazide (PMA). Previously, ethidium monoazide (EMA) was used for discriminating live from dead bacterial cells [6,7]. EMA is a DNA/RNA intercalating substance that only enters bacterial cells with compromised cell walls and cell membranes. However, EMA was reported to possibly to penetrate viable cells of some bacterial species, resulting in underestimation of viable bacterial numbers [8-11]. Because PMA is less able to penetrate viable cells, more attention has been paid to PMA as an alternative to EMA [8]. In the present study, we examined the population of live and dead bacteria in oral specimens. The relationships of cell viability with saliva and dental plaque or carious dentin were further analyzed. Finally, we analyzed the cell viability of *S. mutans* assessed by this PMA technique after treatment with hydrogen peroxide (H_2_O_2_) and proposed the usefulness of this technique for biofilm experiments. This is the first report to apply the combination of PMA plus real-time PCR (PMA-qPCR) for analysis of the prevalence of live/dead *S. mutans* cells in oral specimens and to reveal the relationship between cell numbers in saliva and cell numbers in dental plaque and/or carious dentin. Additionally, we applied PMA-qPCR for monitoring viable *S. mutans* cell numbers *in vitro* in planktonic cells and biofilm treated with various concentrations of H_2_O_2_ for possible application in biofilm experiments.

## Results

### Specificities and sensitivities of the qPCR assay

Fifty-two bacterial strains, including *S. mutans* and *S. sobrinus* strains, were tested using primers designed from genome regions specific for the bacterial strains. Each specific primer pair had broad specificity for the *S. mutans* or *S. sobrinus* strains (Table 1). Standard curves for linear regression between the threshold cycle (Ct) values and corresponding colony-forming units (CFU) were obtained by 10-fold serial dilutions of *S. mutans* and *S. sobrinus* cultures. The regression equations for the standard curves for *S. mutans* and *S. sobrinus* were Y = −2.994X + 35.61 (*R*^2^ = 0.9914) and Y = −3.230X + 37.73 (*R*^2^ = 0.9998), where Y = Ct, X = log_10_x, and x = CFU, respectively (Additional file 1: Figures S1A and S1B). The dynamic ranges were equivalent to 10^2^ to 10^9^ CFU for both *S. mutans* (9.07 × 10^−4^ to 9.07 × 10^3^ μg of chromosomal DNA) and *S. sobrinus* (2.19 × 10^−4^ to 2.19 × 10^3^ μg of chromosomal DNA) per reaction mixture.

**Table 1:** **Strains and amplification results for *S. mutans *and *S. sobrinus***

**Strain**	**Primers used for amplification**		
	***S. mutans*-specific**	***S. sobrinus*-specific**	**Universal**
*S. mutans* UA159	+	-	+
*S. mutans* Xc	+	-	+
*S. mutans* MT703R	+	-	+
*S. mutans* MT8148	+	-	+
*S. mutans* OMZ175	+	-	+
*S. mutans* NCTC10449	+	-	+
*S. mutans* Ingbritt	+	-	+
*S. mutans* GS5	+	-	+
*S. sobrinus* MT8145	-	+	+
*S. sobrinus* OU8	-	+	+
*S. sobrinus* OMZ176	-	+	+
*S. sobrinus* AHT-K	-	+	+

### Effects of PMA and EMA on cell viability

**Figure 1:**
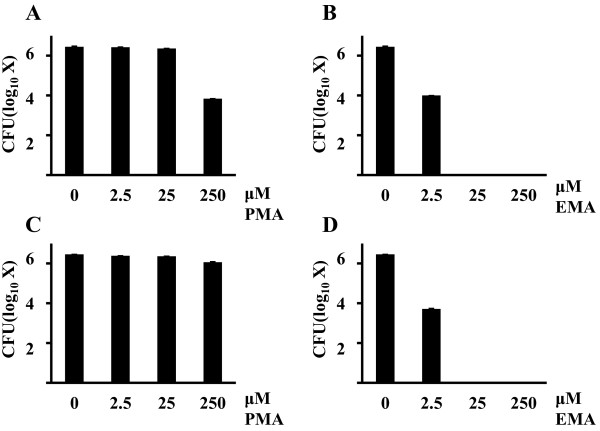
**Effects of PMA (A and C) and EMA (B and D) on *S. mutans *and *S. sobrinus *cell viability.** A total of (**A** and **B**) 2.77 × 10^6^ CFU of *S. mutans* and (**C** and **D**) 2.85 × 10^6^ CFU of *S. sobrinus* were treated with 0, 2.5, 25, and 250 μM PMA and cross-linked. After PMA and EMA treatments, bacterial cells were plated on Mitis-Salivarius agar plates and counted. The mean and S.D. values of independent triplicate data are shown.

We analyzed the effects of various concentrations of PMA on cell viability. The effects of 2.5 and 25 μM PMA on the viability of 2.77 × 10^6^ CFU of *S. mutans* and 2.85 × 10^6^ CFU of *S. sobrinus* were almost the same as that of 0 μM PMA. After PMA treatment, the bacterial cells were counted. The mean (n=3) values for *S. mutans* and *S. sobrinus* were 2.6 × 10^6^ CFU and 2.4 × 10^6^ CFU, respectively, at 2.5 μM PMA; 2.3 × 10^6^ CFU and 2.27 × 10^6^ CFU, respectively, at 25 μM PMA; and 6.77 × 10^3^ CFU and 1.15 × 10^6^ CFU, respectively, at 250 μM PMA. Neither 2.5 or 25 μM PMA treatment had a significant effect on cell viability of either *S. mutans* or *S. sobrinus* (Student’s *t*-test; Figure 1A and 1C), whereas 2.5 μM EMA reduced cell viability of *S. mutans* and *S. sobrinus* by nearly 2.2 log (Figure 1B and 1D). In addition, PCR was not completely inhibited by treatment of dead cells with 2.5 μM PMA (data not shown). Therefore, we used 25 μM PMA in this study.

### Effect of PMA on defined ratios of viable and heat-killed bacterial suspensions

**Figure 2:**
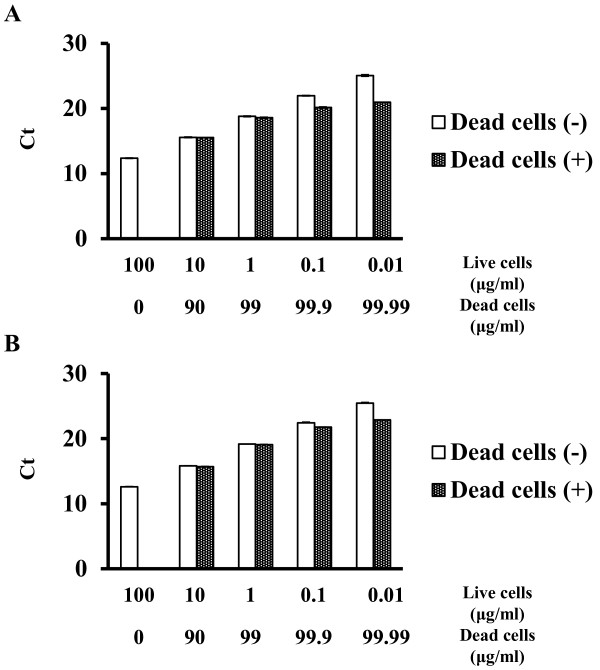
**Effect of 25 μM PMA on heat-killed bacteria as assessed by qPCR.** Serially diluted chromosomal DNA from live cells and live cells spiked with heat-killed cells of (**A**) *S. mutans* and (**B**) *S. sobrinus*. Dead cells (+), *S. mutans*/*S. sobrinus* DNA with DNA from dead *S. mutans*/*S. sobrinus*. Dead cells (−), *S. mutans*/*S. sobrinus* DNA only. All experiments were performed independently three times.

To examine the effectiveness of PMA treatment at selectively detecting viable cells in the presence of dead cells, various mixtures comprising viable and heat-killed cells were evaluated by qPCR. An aliquot each of *S. mutans* and *S. sobrinus* cells was heated at 121°C for 15 min in an autoclave. The heat-killed cells were mixed with untreated original culture cells in defined ratios, with viable cells representing 0.01%, 0.1%, 1%, or 10% of the total bacteria. In both strains, the signals from 0.01 to 100 μg of chromosomal DNA were identical in live cells with and without 25 μM PMA-treated heat-killed cells (Figure 2A and 2B).

### Spiking *S. sobrinus* cells with oral specimens

**Figure 3:**
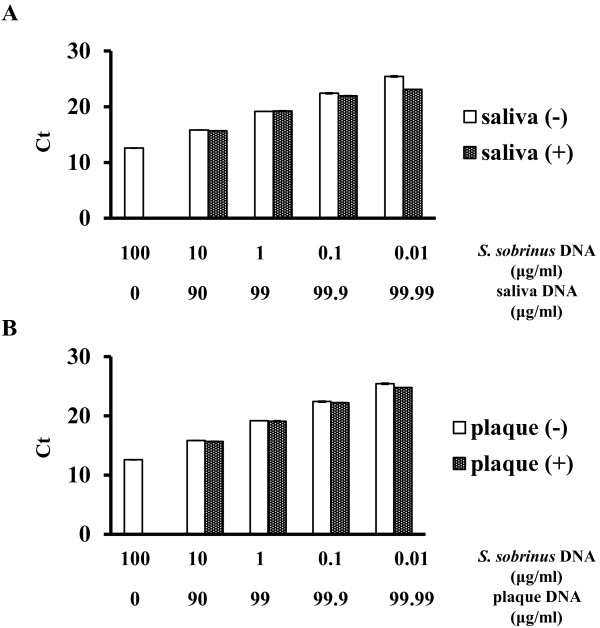
**Effect of oral specimens on qPCR.** Samples of serially diluted *S. sobrinus* chromosomal DNA and *S. sobrinus* chromosomal DNA spiked with DNA from *S. sobrinus*-free oral specimens were analyzed by *S. sobrinus*-specific qPCR. Spike experiments with (**A**) saliva and (**B**) dental plaque. Saliva (+), *S. sobrinus* DNA with DNA from *S. sobrinus*-free saliva. Saliva (−), *S. sobrinus* DNA only. Plaque (+), *S. sobrinus* DNA with DNA from *S. sobrinus*-free dental plaque. Plaque (−), *S. sobrinus* DNA only. All experiments were performed independently three times. Means ± S.D. are shown.

To examine whether PCR was inhibited in the presence of oral specimens, chromosomal DNA from *S. sobrinus*-free saliva and plaque specimens was added to *S. sobrinus* cells. The qPCR analysis of *S. sobrinus* was not inhibited by chromosomal DNA from saliva (Figure 3A) or plaque (Figure 3B).

### Correlation of viable *S. mutans* cell number assessed by PMA-qPCR and by culture

**Figure 4:**
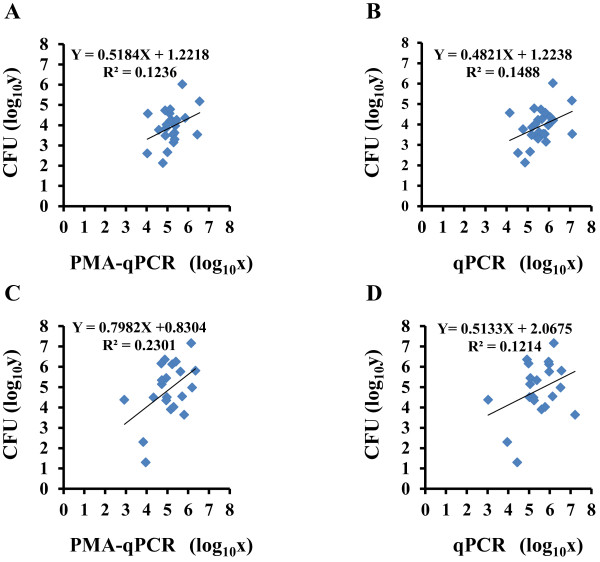
**Correlation between number of viable *S. mutans *cells detected by culture and number detected by PMA-qPCR and/or conventional qPCR.** (**A** and **B**) Dental plaque from caries-free patients (n=24). (**B**) Carious dentin from patients with dental caries (n=21). All data were calculated three times for CFU, PMA-qPCR, and qPCR, and the mean values were plotted. X = log_10_x, where x is the cell number calculated by PMA-qPCR (**A** and **C**) or qPCR (**B** and **D**). Y = log_10_y, where y is CFU.

We compared the *S. mutans* cell number in dental plaque from caries-free patients (n=24) with that from patients with carious dentin (n=21) by qPCR with and without PMA and culture. Positive correlations were observed between the cell number detected by PMA-qPCR and that determined by culture for both caries-free dental plaque (Figure 4A) and carious dentin (Figure 4C). The positive correlations between qPCR and culture are shown in Figure 4B (dental plaque) and 4D (carious dentin). The slopes of the regression equations were lower for qPCR than for PMA-qPCR, indicating that the cell number determined by qPCR was higher than that determined by PMA-qPCR.

### Quantitative discrimination of live/dead cariogenic bacterial cells in oral specimens

**Figure 5:**
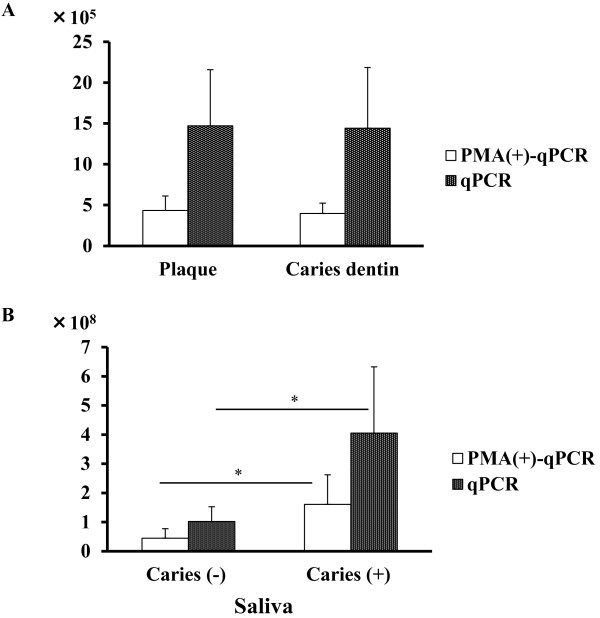
**Comparison of the total (qPCR) and viable (PMA-qPCR) *S. mutans *cell numbers in oral specimens.** (**A**) Dental plaque from caries-free patients (n=24) and carious dentin (n=21). (**B**) Saliva from caries-free children (n=24) and patients with dental caries (n=21). *; p < 0.05

The numbers of *S. mutans* and *S. sobrinus* cells in carious dentin and saliva were quantified in patients with dental caries. As shown in Figure 5A, the mean totals of *S. mutans* cells (±S.D.) calculated by qPCR without PMA were 1.47 × 10^6^ (±6.88 × 10^5^) per 1 mg dental plaque (wet weight) from caries-free donors (n=24) and 1.48 × 10^6^ (±7.80 × 10^5^) per 1 mg carious dentin (wet weight) (n=21); viable cell numbers calculated by qPCR with PMA were 3.98 × 10^5^ (±1.27 × 10^5^) per 1 mg carious dentin (wet weight) and 3.86 × 10^5^ (±1.33 × 10^5^) per 1 mg dental plaque (wet weight), representing 26.9% and 29.5% of the total cells, respectively (Figure 5A). There was no significant difference in viable cell number or total cell number between caries dentin and plaque (Mann–Whitney test).

Next, we compared the number of cells in saliva from patients with and without dental caries. The mean totals of *S. mutans* cells (± S.D.) calculated by qPCR were 4.24 × 10^8^ (±2.38×10^8^) per 1 ml of saliva from patients with dental caries (n=21) and 1.02 × 10^8^ (±5.07×10^7^) per 1 ml of saliva from caries-free donors (n=24); viable cell numbers calculated by qPCR with PMA were 1.68 × 10^8^ (±1.06×10^8^) per 1 ml of saliva from patients with dental caries (n=21) and 4.45 × 10^7^ (±3.31×10^7^) per 1 ml of saliva from caries-free donors (n=24), representing 39.6% and 43.6% of the total cells, respectively (Figure 5B). Total cell number and viable cell number differed significantly between caries-positive and -negative saliva (p < 0.05 for each; Mann–Whitney test).

*Streptococcus sobrinus* was detected in only one patient with dental caries (data not shown). The total numbers of *S. sobrinus* cells calculated by qPCR without PMA were 8.14 × 10^7^ CFU per 1 ml of saliva (32.5% were live cells) and 1.58 × 10^9^ CFU per 1 mg carious dentin (7.84% were live cells).

### Correlation of viable *S. mutans* cell number among oral specimens

**Figure 6:**
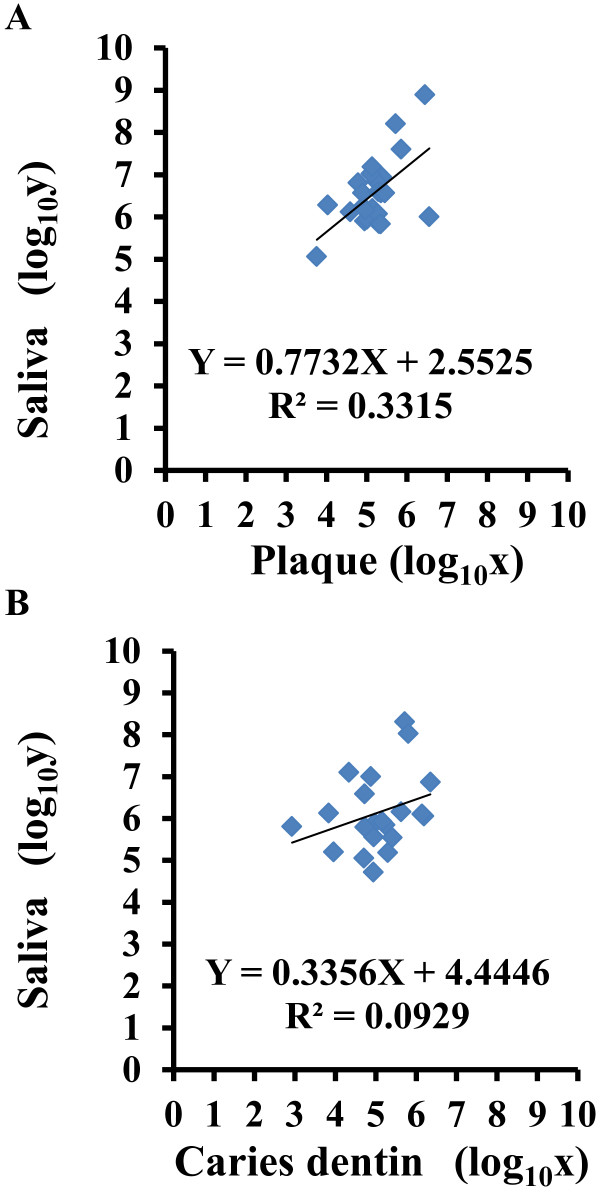
**Correlation of viable *S. mutans *cell number in saliva with viable *S. mutans *cell number in (A) dental plaque from caries-free patients (n=24) and (B) carious dentin (n=21) as assessed by PMA-qPCR.** All data were calculated three times, and the mean values were plotted. X = log_10_x, where x is the viable cell number in dental plaque (**A**) or carious dentin (**B**). Y = log_10_y, where y is the viable cell number in saliva.

The correlations of viable cell number between saliva and caries-free plaque and/or carious dentin were examined. Among caries-free patients, the number of viable *S. mutans* cells in saliva was significantly correlated with the number in plaque (n=24, Figure 6A). No correlation was observed between saliva and carious dentin (n=21, Figure 6B).

### Application of PMA-qPCR for monitoring live bacteria in biofilm and the planktonic phase

**Figure 7:**
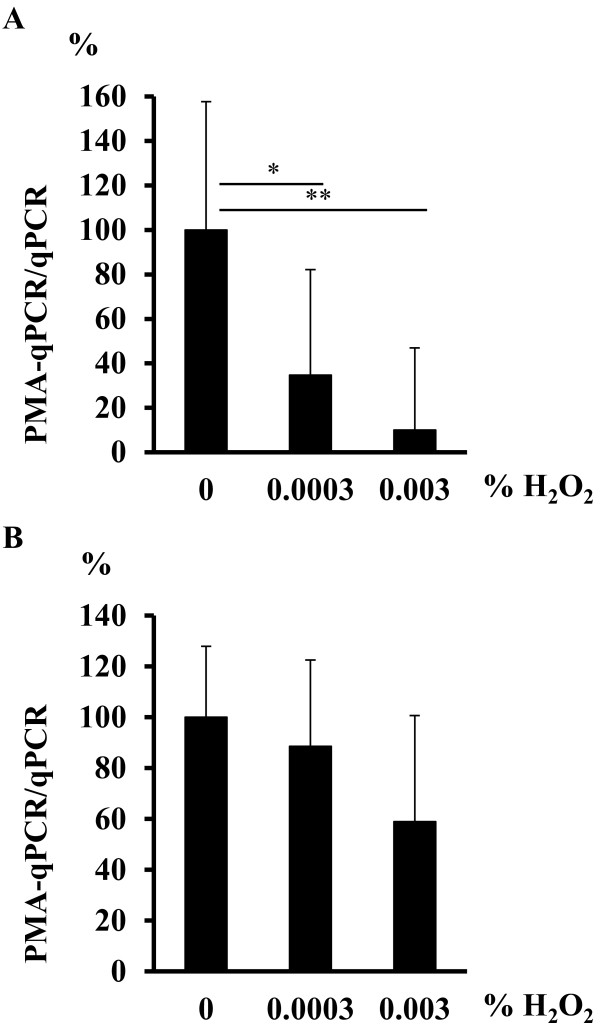
**Monitoring the ratio of viable cell number to total cell number for *S. mutans *in (A) planktonic cells and (B) biofilms, by PMA-qPCR.** Both planktonic cells and biofilms were treated with 0–0.003% H_2_O_2_ for 24 h. The mean ± S.D. values of independent triplicate data are shown. *p < 0.05, **p < 0.01.

One purpose for the development of this assay was to monitor the viable cell number in biofilm. To evaluate the *S. mutans* cell number in both planktonic and biofilm forms, the cells were exposed to various concentrations of H_2_O_2_. In the planktonic phase, the number of viable *S. mutans* cells in 0.0003% H_2_O_2_ was only 10.0% of the number in H_2_O_2_-untreated cells, whereas the number in 0.003% H_2_O_2_ was 34.7% of that in H_2_O_2_-untreated cells (Figure 7A). There was a significant difference in the viable/total cell ratio between 0% and 0.0003% H_2_O_2_ (Bonferroni test; p < 0.05) and between 0% and 0.003% H_2_O_2_ (Bonferroni test; p < 0.01). In biofilm, the number of viable *S. mutans* cells in 0.0003% H_2_O_2_ was 88.6% of the number in H_2_O_2_-untreated cells, whereas that in 0.003% H_2_O_2_ was 58.9% of that in H_2_O_2_-untreated cells (Figure 7B). There was no significant difference in the viable/total cell ratio between 0% and 0.0003% H_2_O_2_ or between 0% and 0.003% H_2_O_2_.

## Discussion

*Streptococcus mutans* and *S. sobrinus* are considered to be cariogenic pathogens in humans [12]. Various studies have monitored the prevalence of caries-related organisms in oral specimens [13]. However, attempts to differentiate between viable and dead bacteria in oral specimens in relation to dental caries have not been reported. In the present study, we initially developed a quantification method for discriminating live and dead cariogenic bacteria, specifically for *S. mutans* and *S. sobrinus*. Previous investigations have reported that EMA has a strong inhibitory effect on the amplification of genomic DNA from viable cells [11], and our study confirmed that EMA itself decreases cell viability. Therefore, all experiments were conducted with PMA, which penetrates a damaged cell membrane and intercalates into DNA, resulting in the inhibition of PCR, in combination with qPCR to quantitatively differentiate between viable and dead cells. We further performed a spiking experiment to evaluate whether this assay was applicable to oral specimens. In general, obtaining oral specimens that do not contain *S. mutans* is challenging, whereas obtaining *S. sobrinus*-free oral samples is relatively easy. For this reason, we performed PMA-qPCR amplification specific for *S. sobrinus* using *S. sobrinus*-free saliva and *S. sobrinus*-free dental plaque as an alternative in the spiking experiment. As shown in Figure 3, neither saliva nor dental plaque inhibited the PCR, indicating that this assay is applicable for measuring cariogenic bacteria in oral specimens.

We next examined the correlation between the numbers of viable *S. mutans* cells in oral specimens as detected by PMA-qPCR and by culture. We found a positive correlation between these quantification methods for both carious dentin and dental plaque. Compared with culture, the number of viable *S. mutans* cells was overestimated by PMA-qPCR. It may be that the culture method usually underestimates the cell number. The cell number determined by conventional qPCR correlated with the cell number determined by culture. Several previous investigations have reported that the cell number determined by qPCR correlated with CFU [14,15]. However, compared with PMA-qPCR, conventional qPCR overestimated the cell number to a greater extent in both types of clinical specimens. Therefore, the cell culture count was closer to the number determined by PMA-qPCR than to that determined by conventional qPCR in the present study.

Monitoring viable bacterial cells in oral specimens provides information to help understand oral infectious diseases. When we compared the total and viable cell numbers in carious dentin from patients with dental caries and dental plaque from caries-free children, there was no significant difference between carious dentin and dental plaque in terms of either total number *S. mutans* cells or number of viable cells. We may not be able to simply compare the cell numbers in these specimens because the contents are not identical. Nevertheless, there was no significant difference in the percentage of viable cells between the specimens. However, there was a significant difference in total cell number and viable cell number between saliva from patients with dental caries and saliva from caries-free children. Monitoring of the viable cell number in relation to the total cell number in oral specimens has not previously been performed. To understand the variation in the viable cell number, both the viable and total cell numbers must be determined. To further understand cell viability in relation to dental caries, a greater number of specimens should be analyzed.

When the relationship between the number of viable *S. mutans* cells in saliva and in dental plaque from caries-free children was analyzed using PMA-qPCR, a positive correlation was found between viable *S. mutans* cells in saliva and in dental plaque. This result was consistent with previous reports [16]. There was no significant correlation between the number of viable *S. mutans* cells in saliva and that in carious dentin from caries patients in the present study. Our data suggest that saliva reflects the number of viable cells in caries-free plaque, but not in carious dentin. Previous studies have found that the microbial biota in the dental plaque of children with severe early-childhood caries was significantly less diverse and less complex than that in caries-free children [17]. Therefore, the viability of cariogenic bacteria in saliva may differ between caries-active and caries-free patients. This possibility should be explored in future studies.

Finally, we evaluated the number of viable of *S. mutans* cells in the planktonic phase and in biofilm. In the planktonic phase, the ratio of viable cells to total bacteria decreased with an increase in H_2_O_2_ concentration (34.7% at 0.0003% H_2_O_2_ and 10.0% at 0.003% H_2_O_2_). There was a significant difference in the viable/total bacterial ratio between 0% and 0.0003 and between 0% and 0.003% H_2_O_2_. However, the decreases in the viable/total cell ratio in biofilm at these concentrations were smaller (88.6% at 0.0003% H_2_O_2_ and 58.9% at 0.003% H_2_O_2_), and there was no significant difference between 0% and 0.0003 or 0.003% H_2_O_2_. These results suggest that PMA-qPCR is applicable for monitoring the numbers of viable and dead cells in biofilm. In biofilm experiments, a live/dead stain is sometimes used to distinguish visually between live and dead bacteria [18]. Although PMA-qPCR is advantageous for quantifying viable cells, it does not provide the visualization obtained with live/dead staining. PMA-qPCR may be a powerful tool for monitoring the number of viable cells in oral biofilms.

## Conclusions

We developed a discriminative quantification method for viable and dead *S. mutans* and *S. sobrinus* cells. We evaluated the potential of this assay and applied it to analyze the prevalence of live/dead cariogenic bacteria in oral specimens and to monitor live/dead cells in biofilm experiments. The ability to discrimination between live and dead bacterial cells in biofilm is essential for studying biofilm, and this assay will be helpful for oral biofilm research. Our assay will contribute to elucidating the role of viable bacteria in oral biofilm and saliva in relation to disease activities.

## Methods

### Reference strains

The 52 reference strains used in the present study were *S. mutans* UA159, *S. mutans* Xc, *S. mutans* MT703R, *S. mutans* MT8148, *S. mutans* OMZ175, *S. mutans* NCTC10449, *S. mutans* Ingbritt, *S. mutans* GS5, *S. sobrinus* MT8145, *S. sobrinus* OU8, *S. sobrinus* OMZ176, *S. sobrinus* AHT-K, *Streptococcus S. downei* Mfe28, *S. downei* S28, *Streptococcus ratti* BHT, *S. ratti* FA1, *Streptococcus cricentus* HS1, *S. cricentus* E49, *Streptococcus mitis* 903, *Streptococcus sanguinis* ATCC 10556, *S. sanguinis* ATCC 10557, *S. sanguinis* OMZ9, *Streptococcus gordonii* DL1, *Streptococcus oralis* ATCC 557, *Streptococcus salivarius* HHT, *Streptococcus anginosus* FW73, *Streptococcus milleri* NCTC10707, *Lactobacillus rhamnosus* JCM1136, *L. rhamnosus* JCM1561, *L. rhamnosus* JCM1563, *L. rhamnosus* JCM8134, *L. rhamnosus* JCM8135, *L. rhamnosus* JCM8135, *Lactobacillus casei* JCM8132, *Porphyromonas gingivalis* W83, *P. gingivalis* 33277, *Treponema denticola* ATCC 35405, *Treponema medium* ATCC 700293, *Treponema vincenti* ATCC 35580, *Treponema pectinovorum* ATCC 33768, *Aggregatibacter actinomycetemcomitans* Y4, *A. actinomycetemcomitans* JCM8577, *A. actinomycetemcomitans* SUNYaB67, *A. actinomycetemcomitans* SUNYaB75, *Aggregatibacter naeslundii* JCM8350, *Prevotella loescheii* JCM8530, *Prevotella denticola* JCM8525, *Prevotella bivia* JCM6331, *Prevotella pallens* JCM1140, *Prevotella veroralis* JCM6290, and *Prevotella oralis* ATCC 33322.

### Ethics statement

All patients were treated in accordance with the Helsinki Declaration regarding the participation of human subjects in medical research. Ethics clearance for the study was obtained from the Ethics Committee of Kyushu Dental University Hospital (reference number 11–40). The parents of participants were fully informed about the study and signed informed consent forms.

### Study subjects and oral specimen sampling

Twenty-one subjects ranging in age from 3 to 10 years and who had dental caries were included in the caries group (mean age ± S.D. = 7.86 ± 0.43 years; 11 males and 10 females). A healthy (completely caries-free) control group consisted of 24 subjects (ages 3 to 12 years; mean age ± S.D. = 7.29 ± 0.56 years; 13 males and 11 females). The carious dentin was excavated from cavitated lesions. Before excavation of the carious dentin, the plaque on the surfaces of cavitated lesions was swiped. The dental plaque samples from healthy subjects were collected from the buccal or lingual surface of the second primary molar. Collected carious dentin and dental plaque were placed in 200 μl of PBS in a sterile 1.5-ml microcentrifuge tube. These samples were washed and placed in PBS solution adjusted to 1 mg per 100 μl. Saliva was collected from both the caries and healthy control groups. Fifty microliters of saliva was washed with PBS and used for analysis.

### Bacterial counting from oral specimens on an agar plate

Serially diluted carious dentin or dental plaque was plated on a Mitis-Salivarius agar plate (Becton Dickinson, Franklin Lakes, NJ) supplemented with 150 g/l sucrose and 200 U/l bacitracin for selection of mutans streptococci (MSB agar). Bacterial counting was performed using a magnifying loupe.

### Propidium monoazide treatment

For only viable cell quantification, PMA (3-amino-8-azido-5-[3-(diethylmethylammonio)propyl]-6-phenyl dichloride; Wako Pure Chemical, Osaka, Japan) treatment was performed for bacterial cells prior to DNA extraction, as previously described [19]. Briefly, PMA was dissolved in 20% DMSO to produce a 25-mM stock solution. Following incubation with the dye for 5 min in the dark, similarly prepared cells were exposed for 5 min to a 500-W halogen light placed 15 cm above 500-μl samples in open microcentrifuge tubes on ice. The toxicity of PMA at 2.5–250 μM to *S. mutans* and *S. sobrinus* was analyzed at 37°C. In the present study, 25 μM PMA was employed for the analysis. All data presented are from triplicate independent cultures and/or biofilms.

### DNA techniques

Routine molecular biology techniques were performed as described by Sambrook *et al*. [20]. Chromosomal DNA was isolated from the bacteria using a Puregene DNA isolation kit (Gentra Systems, Minneapolis, MN). Bacterial chromosomal DNA from oral specimens was isolated using MORA-extract (Cosmo Bio, Tokyo, Japan). Next, 150 μl of lysis buffer was added to the pellet. The lysed bacteria were transferred to a tube with glass beads and heated at 90°C for 10 min. The bacterial mixture was then disrupted using a Mini-Bead Beater (BioSpec Products, Bartlesville, OK) with 0.1-mm-diameter glass beads at 4,800 rpm for 2 min. Thereafter, 200 μl of SDS solution was added and heated at 90°C for 10 min. Next, 400 μl of phenol solution was added and mixed for 1 min. After centrifugation, the aliquot was subjected to ethanol precipitation and dissolved in 20 μl of TE buffer.

### qPCR

To monitor cell numbers, qPCR was performed with *S. mutans*- and *S. sobrinus*-specific primers designed using Primer Express 3.0 software (Applied Biosystems, Foster City, CA). The primers specific for *S. mutans* and *S. gordonii* are shown in Table 2. A universal primer was used for confirmation of the presence of chromosomal DNA (Table 2). For confirmation of primer specificities, conventional PCR was performed using the following thermocycle: 95°C for 5 min, followed by 25 cycles of 95°C for 30 s, 47°C for 30 s, and 72°C for 1 min. Quantification of these cells in oral specimens and *in vitro* biofilm was performed using qPCR with the SYBR green dye to detect the Sm3-15 locus (for *S. mutans*) and Ss6 locus (for *S. sobrinus*) amplicons [5]. Bacterial chromosomal DNA was amplified using LightCycler FastStart DNA Master^PLUS^ SYBR Green I (Roche Diagnostics GmbH, Mannheim, Germany). Each reaction mixture (total 20 μl) contained 5 μl of DNA (10 ng/μl), 4 μl of 5× Master Mix, 2 μl each of forward and reverse primer (500 nM each), and 9 μl of pure water. The mixtures were applied to a LightCycler Capillary (Roche Diagnostics). Amplification and detection of specific products were performed using the LightCycler Carousel-based System (Roche Diagnostics) and the following thermocycle: 95°C for 10 min, followed by 45 cycles of 95°C for 10 s, 58°C for 10 s, and 72°C for 12 s. Dissociation curves were generated using the following conditions: 95°C for 1 min, 55°C for 1 min, and then an increase in temperature from 55.0 to 95.0°C with a heating rate of 0.5°C per 10 s. The melting curves with both primer sets showed a single sharp peak (data not shown). DNA concentrations were calculated based on standard curves obtained using 10-fold serial dilutions of bacterial DNA. All data are shown as the mean of triplicate experiments.

**Table 2:** Oligonucleotide primers used in this study

**Primer**	**Sequence (5’→3’)**	**Locus**	**Reference**
Sm F	GTATGCAGGTGAAGAAGC	Sm3-15	[5]
Sm R	TGGCCACACAATGACCAT		
Ss F	TGGCATTGCCGTAGGAAT	Ss6	[5]
Ss R	TTGTCGACGGGTTCTAGT		
Universal F	TCCTACGGGAGGCAGCAG	16S rRNA	[21]
Universal R	GGACTACCAGGGTATCTA	16S rRNA	

*F* forward primer; *R* reverse primer.

### Preparation of biofilms and planktonic cells

To examine *S. mutans* strains for the ability to form biofilm under various H_2_O_2_ concentrations (serially diluted from 0–3%), the biofilm assay was performed. Bacterial cells were precultured overnight in chemically defined medium (CDM) supplemented with 0.5% sucrose, inoculated into 1 ml of 0.5% sucrose CDM (culture:CDM ratio, 1:50), and then incubated for 24 h under anaerobic conditions at 37°C in polystyrene 24-well plates (Corning, Inc., Corning, NY) with final H_2_O_2_ concentrations of 0–0.03% [22]. The viable cell/total cell ratio in 0% H_2_O_2_ was considered to be 100%.

### Statistics

The Mann–Whitney test and Bonferroni’s test were used to determine statistical significance. A difference was deemed significant at *P* < 0.05.

## Abbreviations

EMA: Ethidium monoazide; PMA: Propidium monoazide; qPCR: quantitative polymerase chain reaction.

## Authors’ contributions

AYo participated in the study design, wrote the manuscript, and was responsible for the overall coordination of the study. AYa and SN performed the microbiological analysis, DNA manipulation, and PMA-qPCR analysis. KMo and KMa performed clinical examinations and sampling of oral specimens. IS and SA conducted statistical analyses. TA supervised this study.

## Supplementary Material

Additional file 1: Figure S1 Standard curves for the qPCR assay were generated by the bacterial cell number and *Ct* value. (A) *S. mutans*. (B) *S. sobrinus*. The mean values of independent triplicate data are shown. Click here for file
