# Supplementary figures and images for: Monitoring the prevalence of viable and dead cariogenic bacteria in oral specimens and in vitro biofilms by qPCR combined with propidium monoazide
Source: BMC Microbiol. 2013 Jul 13;13:157. doi: 10.1186/1471-2180-13-157 (PMC3717283; doi:10.1186/1471-2180-13-157)

## Slide 1
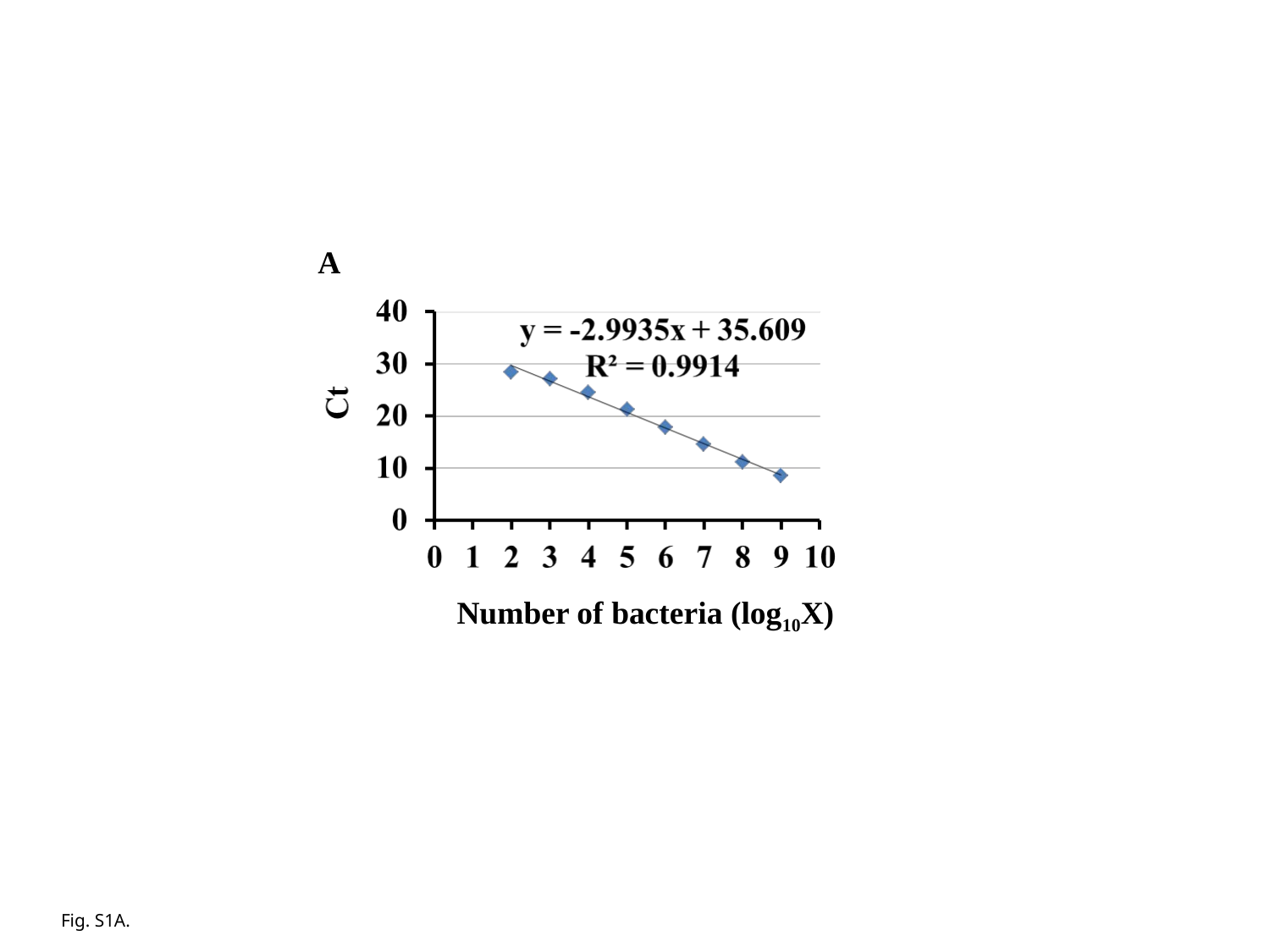

A
Number of bacteria (log10X)
Fig. S1A.

## Slide 2
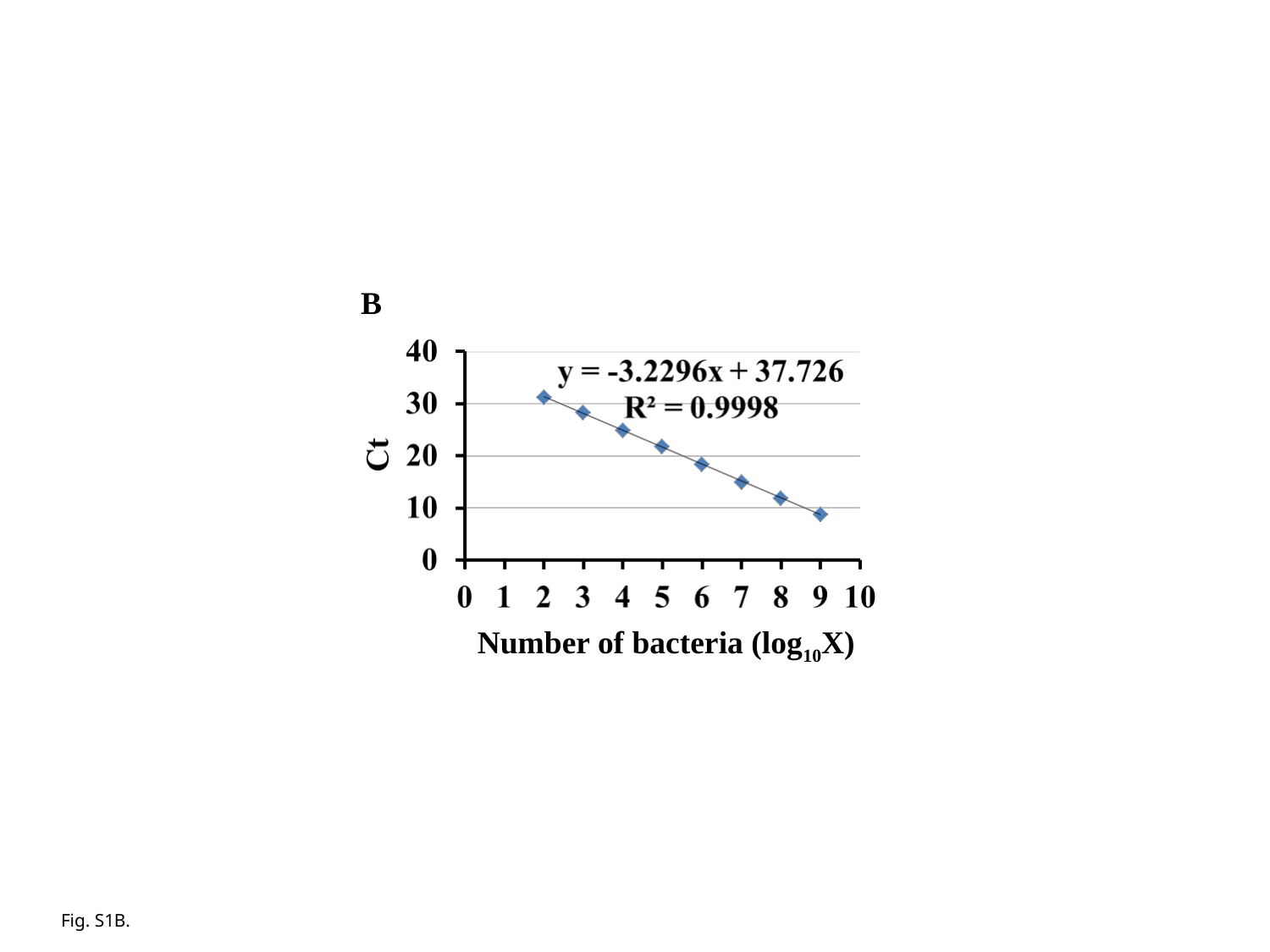

B
Number of bacteria (log10X)
Fig. S1B.

Supplement: Additional file 1: Figure S1 — Standard curves for the qPCR assay were generated by the bacterial cell number and Ct value. (A) S. mutans. (B) S. sobrinus. The mean values of independent triplicate data are shown. [file 1471-2180-13-157-S1.ppt]
